# Recurrence of Retinopathy of Prematurity in Zone II Stage 3+ after Ranibizumab Treatment: A Retrospective Study

**DOI:** 10.1155/2017/5078565

**Published:** 2017-04-09

**Authors:** Qinrui Hu, Yujing Bai, Xiaoli Chen, Lvzhen Huang, Yi Chen, Xiaoxin Li

**Affiliations:** ^1^Department of Ophthalmology, Peking University People's Hospital, Beijing Key Laboratory for the Diagnosis and Treatment of Retinal and Choroid Diseases, Beijing, China; ^2^Department of Ophthalmology, China-Japan Friendship Hospital, Beijing, China

## Abstract

*Objective*. To determine the prevalence and risk factors for the recurrence of retinopathy of prematurity (ROP) in Zone II Stage 3+ after ranibizumab treatment. *Methods*. This was a retrospective, nonrandomized, noncontrolled study that excluded Zone I and aggressive posterior ROP (APROP) cases. Infants who developed Zone II Stage 3 ROP with plus disease and underwent initial intravitreal injection of ranibizumab (IVR) were recruited. Patients were divided into 2 groups based on the outcome after initial ranibizumab treatment: recurrence of ROP or favorable outcome. Data was collected and analyzed by SPSS 16.0. *Results*. Forty-two patients were included, and 80 eyes with Zone II Stage 3+ were subjected to IVR treatment. Eleven of 42 patients (26.2%, 18 eyes) had a recurrence of ROP after the initial treatment. On univariate analysis, preretinal hemorrhage before treatment was significantly different between the two groups (*P* = 0.000). Multivariate analysis found that preretinal hemorrhage before treatment was the only factor associated with the recurrence of ROP in our study (*P* = 0.004). *Conclusions*. The recurrence rate of ROP in Zone II Stage 3+ after initial ranibizumab treatment was notable and preretinal hemorrhage before treatment was associated with the recurrence of ROP in our study.

## 1. Introduction

Retinopathy of prematurity (ROP) is a neovascular disorder that occurs in premature infants, and it continues to be a major cause of preventable childhood blindness all over the world [1, 2]. Because of the availability of high-quality neonatal intensive care, neonatal mortality has declined considerably in many countries. However, the improved survival of very premature infants has also resulted in an increased rate of ROP that requires close screening and treatment [3]. Vascular endothelial growth factor (VEGF) is an important factor in the pathologic angiogenesis of ROP, and blocking the action of VEGF might be expected to reduce vascular activity. Hence, an anti-VEGF strategy may be a promising prospect in the current treatment of ROP. Anti-VEGF drugs have been gradually introduced because they possess the advantages of fewer risks from general anesthesia in physically compromised infants, require a less time-consuming procedure, and potentially have a lower chance of unfavorable outcomes.

However, ranibizumab treatment may be associated with a higher incidence of reactivation when compared with bevacizumab treatment in ROP [4]. The anti-VEGF drugs also show comparable or inferior results to conventional laser therapy in treatment of Zone II ROP. Recurrence rates vary greatly, especially based on the drugs and doses used and whether aggressive posterior ROP is being treated [4–7]. Several studies have explored the association of both prenatal and postnatal factors with the progress and treatment of ROP [8–10]. Less well understood are the mechanisms and the susceptible risks for recurrence of ROP. Only a few reports have focused specifically on this problem [11]. In the present study, we examined the clinical features and risk factors associated with the recurrence of ROP in Zone II Stage 3+ after ranibizumab treatment.

## 2. Methods

### 2.1. Data Collection

The study was conducted in our Department of Ophthalmology, which is one of the ROP referral centers in North China. All babies less than 32 weeks gestational age or with a birth weight of less than 2000 g should be screened for ROP. Babies born with risk factors for ROP should also be screened. The medical records of infants who met the criteria for ROP screening and developed Zone II Stage 3 ROP with plus disease from January 2014 to September 2015 were retrieved.

After informed consent was obtained, an intravitreal injection of 0.25 mg/0.025 mL ranibizumab (IVR; Lucentis, Novus, US) was given to each patient. The inclusion criteria were as follows: (1) Zone II Stage 3 ROP with plus disease which received IVR as the initial treatment and (2) completed all of the follow-up visits. The study excluded Zone I and aggressive posterior ROP (APROP) cases. The patients were divided into 2 groups based on the recurrence of ROP after the initial ranibizumab treatment. The data collected from the infants' records included gender, birth weight, gestational age, multiple birth (twins or triplets), respiratory distress syndrome, sepsis, hypoxic ischemic encephalopathy (HIE), anemia, pneumonia, hypotension, carbohemia, intraventricular hemorrhage, blood transfusion, oxygen exposure, surfactant administration, the use of vitamin E, and the use of hormones. Factors in pregnancy included preeclampsia, placental abruption, and intrauterine hypoxia.

### 2.2. Eye Examination, Monitoring, and Management

Senior ophthalmologists identified the location and sequential retinal changes of ROP and performed all examinations. Patients' pupils were dilated with 0.5% tropicamide and 0.5% phenylephrine drops 2 h before examination. Indirect ophthalmoscopy was routinely performed with the use of a lid speculum and scleral indentation after topical anesthesia. Digital retinal images were also obtained with RetCam for the objective documentation of retinal findings. ROP findings included the preoperative ROP zone and stage, preretinal hemorrhage (at both pretreatment and posttreatment), iris neovascularization, recurrence time, and therapy method.

Examinations were performed on a weekly or biweekly basis, depending on the retinal findings, and continued until vascularization had reached Zone III or established ROP was definitely regressing. The recurrence was characterized by any worsening signs, including an aggravated ridge or plus signs after the initial regression. For the late reactivation of ROP, termination of the examination took place when the far peripheral retina was not vascularized. An examination was also required until the vascularization had reached an acceptable level (within 1 disc diameter of the ora serrata).

Statistical analysis was performed to compare the 2 groups with statistical software (StatLab, SPSS for Windows, version 16.0; SPSS Inc., Chicago, Illinois, USA). Univariate analysis to determine the association between risk factors and the recurrence was performed via a *t*-test or a chi-square test. Logistic regression analysis was performed to explore the relationship between recurrence and suspected risk factors with the removal criterion of 0.50.

## 3. Results

Forty-six patients (24 females, 22 males) underwent an initial IVR treatment. Among them, the disease regressed in 4 patients (8 eyes, 9.1%) within one week after treatment, and these patients returned to their local primary hospitals to complete their follow-up. We excluded them owing to the lack of data from the last visit. Eighty eyes of 42 patients belonging to Zone II Stage 3+ ROP accepted IVR treatment, and 4 eyes were classified as Zone I Stage 2+, Zone II Stage 2+ ROP, or healthy. Of the 42 patients, 20 were females and 22 were males, with a mean gestational age of 29.4 ± 2.2 weeks (range: 26.3–37.1 weeks) and a mean follow-up of 67.9 ± 15.5 weeks by adjusted age (gestational age and postnatal age).

**Figure 1:**
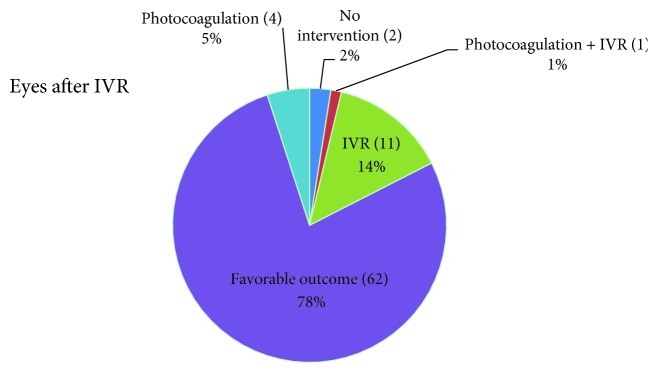
Results and secondary therapies of 80 eyes of infants with retinopathy of prematurity after initial ranibizumab treatment (IVR).

Eleven of the 42 patients (26.2%; 18 out of 80 eyes, 22.5%) had a recurrence of ROP after the initial IVR treatment (Figure 1). The mean treatment interval to recurrence was 8.5 ± 5.7 weeks after the treatment with a mean adjusted age of 45.7 ± 6.1 weeks. Sixteen out of the 18 eyes required an additional intervention, including a second intravitreal injection (11 eyes, 61.1%), photocoagulation (4 eyes, 22.2%), or combination therapy (1 eye, 5.6%). Two eyes (11.1%) regressed spontaneously without a second intervention. Retinal detachment was not observed. The hemorrhage mainly occurred around the ridges of the temporal side at an average of 3.1 ± 2.6 disc diameters. Hemorrhage occurred in two eyes at posterior Zone I and covered the macular area of more than 5 disc diameters.

Per univariate analysis, there was a significant difference between the two groups in preretinal hemorrhage before treatment (*P* = 0.000). The data and statistics are summarized in Table 1. The results of the logistic regression analysis showed that preretinal hemorrhage before treatment was associated with the recurrence of ROP (Table 2).

## 4. Discussion

Our study found that the rate of recurrence for ROP in Zone II Stage 3+ was 26.2%, and the recurrence took place at an average of 8.5 weeks after the initial ranibizumab treatment. Preretinal hemorrhage before treatment was an important risk factor that was associated with the recurrence of ROP.

Recurrence of ROP is a serious problem that can produce severe outcomes, such as vitreoretinal traction and retinal detachment. This study was designed to explore the possible contributing factors and therapy strategy for the recurrence of ROP with IVR.

Eleven patients (26.2%) in our study treated with ranibizumab had a reactivation of ROP after the initial response to the treatment. A previous study reported that of 425 eyes treated with ranibizumab in Zone II, 144 (31.0%) eyes had a recurrence of ROP and required additional treatment [12]. This difference might be explained by a lower sample size in the present study; in addition, the previous study recruited patients with several stages of ROP (e.g., aggressive posterior ROP) for the analysis. There is an increasing body of evidence that suggests that IVR is associated with a higher recurrence compared to IVB in the treatment of patients with ROP. Recently, a study demonstrated an 83% higher rate of relapse after ranibizumab treatment, and eventually, all the eyes required supplemental laser therapy [13]. Another study found that the incidence of disease relapse was higher in eyes that received ranibizumab compared with bevacizumab in the treatment of type 1 ROP [14]. One concern is the differential effect on recurrence brought about by the two drugs. One case report found that serum VEGF levels in an infant treated with ranibizumab were suppressed for 3 weeks and returned to original levels 4 weeks later [15]. A much longer systemic half-life had been noted with bevacizumab than that with ranibizumab in adult patients (20 days versus 2 hours) [4]. The treatment interval to reactivation time was 16 weeks in the BEAT-ROP study with bevacizumab [7]. A short half-life and a low systemic absorption may decrease possible side effects, and the rapid clearing of the vitreous and serum levels of VEGF often suggests a high chance of recurrence. These factors can explain why the average treatment interval of 8.5 weeks in our study was earlier than that of other cases treated by bevacizumab. In our study, patients were administered a dose of 0.25 mg/0.025 mL ranibizumab. The injected volume and the use of ranibizumab may have contributed to recurrence for a variety of reasons. Therefore, further investigation is required to find an optimal combination for different stages of ROP.

We identified that preretinal hemorrhage before treatment was a risk factor that was associated with the recurrence of ROP in specific Zone II Stage 3+ after ranibizumab treatment. Our study was concerned with hemorrhage, which is a common phenomenon in patients. Twenty-two out of 80 eyes (27.5%) presented with hemorrhage before treatment. A hemorrhage incidence of 22% was reported in the study by Pollack et al., which was comparable to our findings, but was much lower than the studies by Watts et al. that described an incidence of 54.2% [5, 9, 16, 17]. A retrospective study suggested that both pre- and posttreatment hemorrhages (retinal or vitreous) were significantly associated with the progression to retinal detachment along with vitreous organization [5]. Hemorrhage usually causes surface wrinkling or traction and macular detachment due to the progressive fibrovascular proliferation and contraction along the posterior surface of the vitreous [10, 18]. A preretinal hemorrhage with a small disc diameter at the shunt may resolve spontaneously, while one with a large disc diameter in the posterior pole may be permanent. It is reasonable to infer that the hemorrhage may contribute to abnormal vitreous organization and a high recurrence of ROP and retinal detachment.

In our study, 11 out of the 18 relapsed eyes (66.1%) required secondary ranibizumab treatment and 4 eyes (22.2%) received photocoagulation. Only one case received combination therapy. Photocoagulation was the priority recommendation for the treatment of recurrence due to fibrovascular membrane proliferation. For slowly progressing recurrence with a degree lower than that of baseline, we recommended IVR as the supplemental treatment. The study implied that IVR was a valuable treatment option for the management of the recurrence of ROP in Zone II, in consideration of the structural damage and adverse long-term outcomes associated with laser treatment. However, one study recently found that fluorescein angiography revealed a scalloped regression pattern in eyes treated with an anti-VEGF drug. The presence of that pattern in conjunction with chronic vascular arrest and peripheral retinal ischemia (in nearly 90% of eyes) that persisted beyond standard screening timelines has significant implications for the management of ROP [19]. This is a new finding worthy of further study. In our study, we did not find any anesthesia accident, respiratory distress, or death in the infants during the follow-up period. To develop a better treatment strategy, it is necessary to evaluate the long-term safety of ranibizumab via fluorescein angiography, especially in patients who received the drug treatment more than once.

Our study was limited as a retrospective and nonrandomized controlled trial. There was also a lack of fluorescein angiography in the evaluation of ROP patients. The recurrence rate with IVR remains high for ROP in Zone II. Caution needs to be exercised when using IVR monotherapy because it necessitates a longer follow-up period and a higher rate of secondary intervention compared to conventional laser treatment.

## Figures and Tables

**Table 1:** Univariate analysis of baseline demographics and recurrence in the eyes with retinopathy of prematurity in Zone II Stage 3 after ranibizumab treatment.

	Recurrence (18 eyes)	Favorable outcome (62 eyes)	*P* value
Baseline characteristics			
Patients	11	31	
Birth weight (g)	1204.09 ± 321.36	1356.61 ± 505.10	0.311^a^
Gestation age (weeks)	28.82 ± 1.36	29.72 ± 2.39	0.112^a^
Male	5	17	1.000^b^
Multiple gestation	2	11	0.713^b^
Respiratory distress syndrome	4	11	0.481^b^
Sepsis	0	1	1.000^b^
HIE	1	0	0.262^b^
Anemia	4	12	0.720^b^
Pneumonia	1	6	1.000^b^
Hypotension	1	1	0.387^b^
Carbohemia	1	1	0.387^b^
Intraventricular hemorrhage	2	8	1.000^b^
Blood transfusion	2	8	1.000^b^
Oxygen administration	6	20	1.000^b^
Surfactant	0	7	0.654^b^
Vitamin E	1	2	1.000^b^
Hormones	1	0	0.262^b^
Factors during pregnancy			
Preeclampsia	2	2	0.558^b^
Placental abruption	0	4	0.330^b^
Intrauterine hypoxia	1	3	0.558^b^
ROP findings			
Preretinal hemorrhage before treatment	5	6	0.000^b^
Preretinal hemorrhage after treatment	0	2	0.216^b^
Iris neovascularization	1	0	0.349^b^
Threshold	17	52	0.254^b^

^a^Independent samples *t*-test.

^b^Chi-square test.

SD: standard deviation; HIE: hypoxic ischemic encephalopathy; ROP: retinopathy of prematurity.

**Table 2:** Multivariate logistic regression analysis of risk factors for the recurrence of retinopathy of prematurity.

Factor	Odds ratio	*P* value
Birth weight < 1250 g	0.823	0.860
Gestation age < 29.5 weeks	1.332	0.791
Respiratory distress syndrome	5.232	0.221
HIE	3.856E5	0.999
Hypotension	0.000	1.000
Carbohemia	0.000	1.000
Placental abruption	0.000	0.999
Preretinal hemorrhage before treatment	39.404	0.004
Preretinal hemorrhage after treatment	0.000	0.999
Iris neovascularization	7.025E8	1.000
Threshold	0.773	0.883

HIE: hypoxic ischemic encephalopathy.
